# Impact of COVID-19 on the Gut Microbiome: A Review

**DOI:** 10.7759/cureus.91470

**Published:** 2025-09-02

**Authors:** Andrea Pedraza, Sabrina Bonnice, Michelle N Won, Marc M Kesselman, Michelle Demory Beckler

**Affiliations:** 1 Medical School, Nova Southeastern University Dr. Kiran C. Patel College of Osteopathic Medicine, Fort Lauderdale, USA; 2 Medical School, Nova Southeastern University Dr. Kiran C. Patel College of Osteopathic Medicine, Davie, USA; 3 Rheumatology, Nova Southeastern University Dr. Kiran C. Patel College of Osteopathic Medicine, Fort Lauderdale, USA; 4 Microbiology and Immunology, Nova Southeastern University Dr. Kiran C. Patel College of Allopathic Medicine, Fort Lauderdale, USA

**Keywords:** covid 19, covid 19 gi manifestation, covid-19 transmission, gut dysbiosis, long-covid-19, microbiome, probiotics, sars-cov-2

## Abstract

Coronavirus Disease 2019 (COVID-19) has resulted in over 6 million deaths worldwide in fewer than four years and is a result of infection with severe acute respiratory syndrome coronavirus 2 (SARS-CoV-2). The protein that mediates SARS-CoV-2 host cell entry is the angiotensin-converting enzyme 2 (ACE2), which is highly expressed on the membrane of gastrointestinal (GI) cells. Consequently, infection can lead to direct damage to the GI tract and gut dysbiosis, which is associated with an imbalance of microbiota, inflammation, and other systemic infections and diseases. In this review, we will focus on the impact of COVID-19 on the GI system. We will examine the pathophysiology of gut dysbiosis in COVID-19 patients, as well as emphasize the significance of probiotics in addressing this condition. Additionally, we will identify key areas of interest that warrant further investigation.

## Introduction and background

The COVID-19 pandemic made headlines starting in December 2019, causing severe acute respiratory illness and leading to increased morbidity and mortality worldwide [1]. Severe acute respiratory syndrome coronavirus 2 (SARS-CoV-2) entry into host cells is mediated by angiotensin-converting enzyme 2 receptors (ACE2R). This receptor is widely expressed throughout the body but has high expression levels in the respiratory and gastrointestinal (GI) tracts [2]. While respiratory symptoms of infection have been the focus of much research due to their prevalence and severity, the impact of COVID-19 and the gut is an emerging area of research. Jin and colleagues studied 651 COVID-19 patients on admission and found that up to 11% of patients experienced GI disturbances such as nausea, vomiting, and diarrhea prior to respiratory manifestations. Interestingly, patients with severe COVID-19 infection reported more extreme GI symptoms compared to patients with mild infection [3].

The gut microbiota are well known for various benefits on health, including disease protection from pathogenic microbes, reducing inflammation, and promoting immune system function. Recent studies have shown that after the resolution of infections such as SARS-CoV-2, patients can develop gut dysbiosis, defined as a disruption in the microbiome and imbalance of microbiota, even in cases where there is an absence of GI manifestations [4]. GI dysbiosis is associated with local and systemic alterations. These include increases in GI inflammatory responses, viral and bacterial infections, and even malnutrition, as well as more systemic complications such as metabolic disorders, obesity, and lung homeostasis via the gut-lung axis [4,5]. The aim of this review is to examine GI manifestations concomitant with SARS-CoV-2 infection as well as the transmission and pathogens involved, and the efficacy of prebiotic, probiotic, and synbiotic use in prevention and treatment. Emerging therapies will also be discussed.

## Review

Methods

Study Design 

Between September 10 and October 15, 2022, a literature review was conducted following the Preferred Reporting Items for Systematic Reviews and Meta-Analysis (PRISMA) guidelines to review and assess the effects of SARS-CoV-2 on the gut microbiome and how it may be a potential cause of gut dysbiosis. Ethical and Institutional Review Board (IRB) approval was not required. The literature review was based exclusively on published literature. The review did not receive funding. 

Inclusion and Exclusion Criteria

We utilized the Population, Intervention, Comparison, and Outcomes (PICO) tool to determine inclusion and exclusion criteria for the article search. For the population, we included patients of all genders older than 18 years of age, with an established history of COVID-19 infection or current COVID-19 infection. These papers were in the English language. We decided to exclude animal models, systematic reviews, literature reviews, Spanish articles, studies with patients with several comorbidities, and other studies that did not follow our inclusion criteria. We excluded articles older than 4 years. For intervention, the study included subjects with active or past COVID-19 infection who had not received external treatments that could alter gut dysbiosis, such as antibiotics or other remedies. As a comparison, the study aimed to investigate the effects of COVID-19 on the human gut microbiome versus in patients with no previous COVID-19 infection. This study also compared patients with past COVID-19 infections who received prebiotic and probiotic treatment versus those who did not. Finally, as for outcomes, it included a correlation between gut dysbiosis and previous COVID-19 infection, along with improvement of gut dysbiosis with the use of prebiotics/probiotics in patients with previous infection.

Search Strategy and Screening

Between September 10 and October 15, 2022, a search was conducted and completed on four databases: PubMed, CINAHL, EMBASE, and Web of Science. Relevant search terms were run through electronic databases to extract published articles reporting gut dysbiosis in patients with active and previous SARS-CoV-2 infection. All articles identified were uploaded into the Rayyan software (www.rayyan.ai) by a group of three researchers (S.B., M.W., and A.P.). Duplicates were eliminated, and the remaining articles that did not meet the inclusion criteria were removed following the PRISMA criteria. Forty-two articles were screened by our three researchers, five of which were excluded.

Thirty-seven articles underwent a quality review and were labeled as either “pathophysiology”, “transmission”, or “prebiotic/probiotic use”. The labeling for these articles came from the relevance of each study to one of the above-mentioned topics. These were the essential topics that were to be included in the literature review, and sufficient evidence was needed for each. 16 articles were labeled as “pathophysiology”, 11 articles were labeled as “transmission”, and 10 articles were labeled as “prebiotic/probiotic use”. From these 37, a total of 30 studies were included in the final literature review, which was reviewed by all researchers. Five articles were excluded due to either being a systematic review (4), wrong objective (2), or wrong language (1). 

Results

Several clinical trials have evaluated the effects of probiotics, prebiotics, synbiotics, and symbiotics on the gastrointestinal effects of COVID-19. Table 1 provides an overview of these studies discussed, including the treatments, outcome measures, study year, and populations evaluated.

**Table 1:** Treatments discussed and outcomes measured Clinical trials evaluating the gastrointestinal effects of COVID-19: treatments, outcome measures, study year, and populations. Created by the authors based on published literature.

Treatment	Outcome Measure	Study	Year	Population
Dietary one capsule with Lactobacillus K8 3 x 109cfu daily for 2 months	COVID-19 prevention in healthcare workers	Chen, J., & Vitetta et Al. [22]	2021	> 20 years
Sivomixx®	Symptoms Remission	Hung, Y.-P. et Al. [27]	2021	> 18 years
OMNi-BiOTic 10 (Synbiotic by OMNi-BiOTic, Port Chester, USA) Lactobacillus acidophilus W55 Lactobacillus paracasei W20 Lactobacillus acidophilus W37 Lactobacillus rhamnosus W71 Lactobacillus plantarum W1 Lactobacillus salivarius W24 Bifidobacterium lactis W51 Lactobacillus plantarum W62 Enterococcus faecium W54 Bifidobacterium bifidum W23 Other ingredients: Non-GMO (genetically modified organism) corn starch, maltodextrin, inulin, potassium chloride, plant protein (rice), magnesium sulfate, fructooligosaccharides (FOS), amylase, vanilla flavoring, manganese sulfate.	Microbiome Composition	Medical University of Graz [29]	2024	>18 years
Fecal Microbiota Transplantation	Microbiome Composition	Liu et Al. [30]	2021	>18 years

Discussion

Pathophysiology of COVID-19-Associated Gastrointestinal Distress

**Figure 1:**
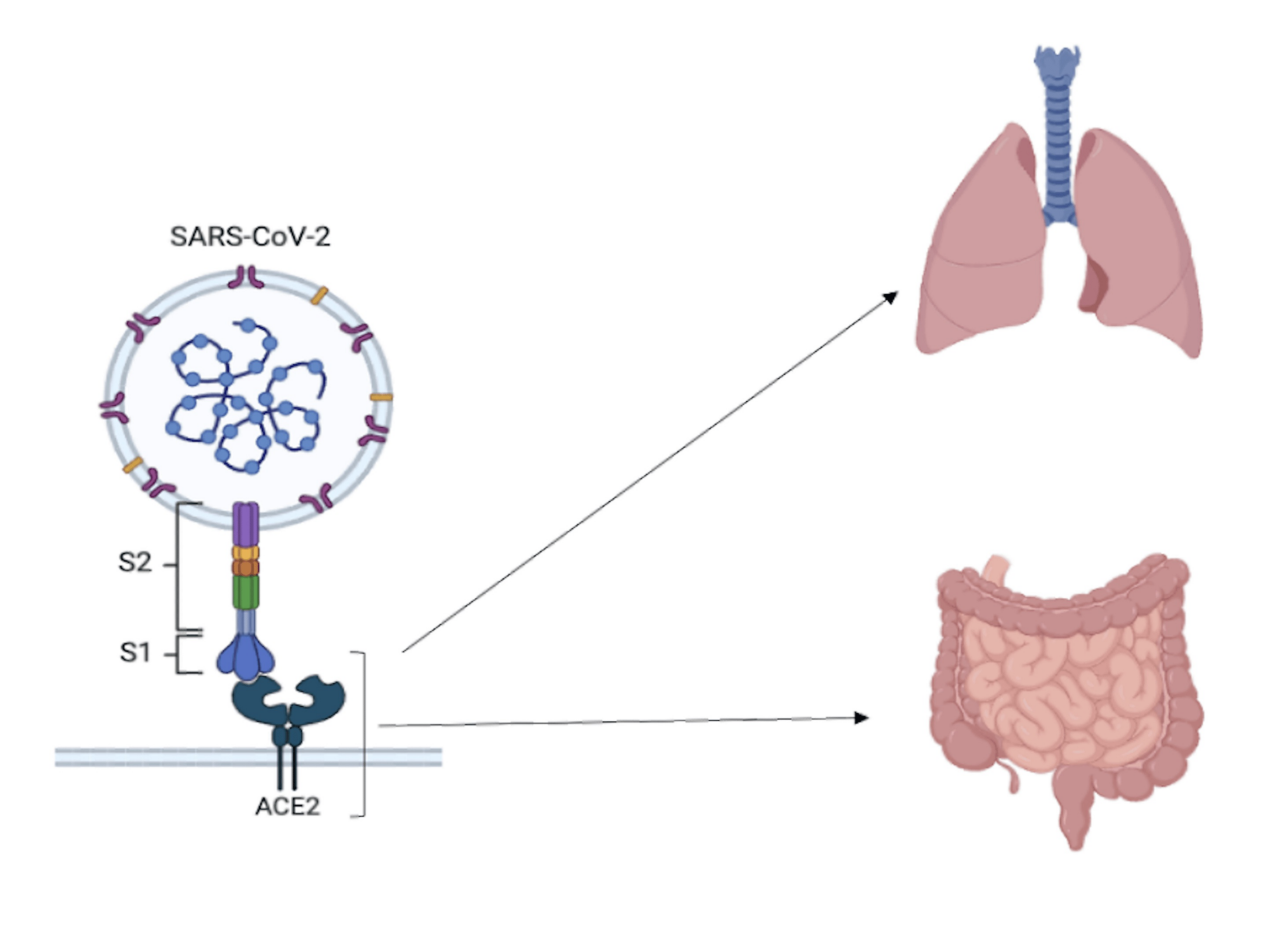
ACE2R in the lungs and gut. ACE2: angiotensin-converting enzyme II; ACE2R: ACE2 receptors; S1: S1 subunit, contains the receptor-binding domain (RBD) that mediates viral attachment to the host ACE2R; S2: S2 subunit, facilitates membrane fusion and viral entry Image created with BioRender (www.biorender.com; BioRender, Toronto, Canada).

In recent years, angiotensin-converting enzyme II receptors (ACE2R) have been identified as important mediators of SARS-CoV-2 entry into respiratory and gastrointestinal mucosa [6]. Figure 1 illustrates the ACE2R in the GI system. While less common than the respiratory symptoms, gastrointestinal distress, including nausea, vomiting, diarrhea, and abdominal pain, has been widely reported in COVID-19 patients all along the clinical spectrum [7]. Emerging evidence indicates that the virus can directly infect the digestive tract, with viral RNA detected in stool samples of infected individuals [8,9]. Importantly, these gastrointestinal symptoms may precede or occur in the absence of respiratory symptoms, so they may be an important clinical indicator of suspected COVID-19 infection [10].

While the exact mechanism of gastrointestinal distress is still under investigation, it is believed to result from both the direct cytopathic effect of the virus on intestinal epithelial cells, as well as indirect immune-mediated injury. Viral entry via ACE2R in intestinal enterocytes, colonocytes, and gastric glandular cells causes significant cellular and molecular damage to the cell [11]. Upon entering the cell via ACE2R, an antiviral inflammatory response is activated, triggering epithelial cells to release interferons, interleukins, and tumor necrosis factor-alpha (TNF-ɑ) [12]. This immune response can lead to gastrointestinal symptoms such as nausea and vomiting [7,8,13]. ACE2 also plays a role in maintaining intestinal barrier integrity and disruption results in increased intestinal permeability and inflammation [11]. Intestinal enterocyte infection with SARS-CoV-2 leads to direct damage to tight junctions, further impairing the gut barrier and may cause diarrhea [9]. 

Furthermore, pyrin domain-containing protein 3 (NLRP3) inflammasome activation is another mechanism by which SARS-CoV-2 may induce gastrointestinal injury. This inflammasome is the most extensively discussed inflammatory complex in relation to the lungs and gut. Upon activation, it triggers the release of proinflammatory cytokines IL-18, IL-6, and IL-1β, which subsequently induce inflammation and subsequent cell death [11]. Recent studies have demonstrated that SARS-COV-2 stimulates NLRP3 activation directly via ORF3a during inflammasome activation, further boosting pro-IL1β and pro-Caspase-1 levels [11,14]. Additionally, SARS-CoV-2 viroporin N and components of the envelope protein both accelerate the activation of the NLRP3 inflammasome, independent of reactive oxygen species (ROS)formation and K+ efflux [11]. NLRP3 is an important determinant of immune response during early phases of infection, and varying levels of this response likely contribute to the clinical heterogeneity of gastrointestinal distress amongst COVID-19 patients [11].

COVID-19 and Gut Microbiome

Gut dysbiosis can occur through three main mechanisms of action, which include a decrease in beneficial bacteria, an increase in opportunistic pathogens, and a decreased gut microbiome diversity. It has been seen that these three mechanisms can occur all at the same time [15]. Gastrointestinal symptoms such as vomiting and diarrhea have been associated with COVID-19 and may be linked to alterations in the gut microbiome. Previous studies have shown that 3.4% to 11.4 % of patients have these gastrointestinal symptoms, especially in critically ill patients [16]. Overall, in a healthy individual, microbes that hold a beneficial role include the major phyla of Firmicutes and Bacteroidetes. *Helicobacter *is the most common type of opportunistic bacteria in the stomach, and this helps determine the entire microbial landscape of the gastric flora [17].

*Lactobacillus *is a common microbe throughout the stomach, colon, and small intestine that provides antimicrobial protection through its production of lactic acid [17]. *Bifidobacterium *plays a large role in the gut by inhibiting pathogens through the production of antibacterial properties and T regulatory responses [18]. *Faecalibacterium prausnitzii *is one of the highly active members of the gut microbiota and plays an important role in intestinal homeostasis [19]. As the severity of GI symptoms increases, there is a direct link to decreased gut microbiome diversity throughout the GI tract [20].

Firmicutes have been shown to be significantly decreased in the COVID-19 patients compared to the control group. Patients with more severe symptoms had lower levels of Firmicutes compared to those with mild or no symptoms. A significantly low ratio of Firmicutes to Bacteroidetes was seen with COVID-19 patients; this is significant for gut dysbiosis. Low ratios of Firmicutes to Bacteroidetes were seen with cases of low-grade systemic inflammation and Crohn's Disease. This microbial interaction affects inflammatory mediators such as TNF-ɑ, interferon-gamma (INF-γ), and interleukin-21 (IL-21), which play a role in the severity of COVID-19 infections and can impact the formation of a cytokine storm. Firmicutes and Bacteroidetes are also linked to the regulation of ACE2 expression in rodents, altering the amount of receptor expression. Overall, there is an activation of macrophages and monocytes, which produce a complex array of pro-inflammatory cytokines [20].

Different studies demonstrated the growth of unusual microorganisms and depletion of common gut microbes in COVID-19 patients. *Helicobacter pylori *(*H. pylori*) is known to increase the expression of ACE2R in the GI tract. This overexpression of ACE2R in the GI tract has been linked to greater severity and duration of infection and may contribute to immune dysregulation [21]. The overexpression of ACE2R observed in the presence of *H. pylori *may facilitate increased entry of SARS-CoV-2 through enterocytes. Initially, among patients who had received a positive result on the PCR test for COVID-19, an *H. pylori *stool antigen test was conducted to distinguish individuals with both COVID-19 and *H. pylori *from those with COVID-19 but without *H. pylori *[21]. Patients were grouped into four groups depending on the severity of the disease.

The four groups included: mild with no hospitalizations or pneumonia; moderate, who were hospitalized but had no pneumonia; moderate/severe, who were hospitalized with pneumonia; and severe patients who were admitted to the ICU due to difficulty breathing from pneumonia. In addition, all the patient's comorbidities were taken into account. Patients positive for both COVID-19 and *H. pylori *had worse abdominal pain and diarrhea compared to those without *H. pylori *[21].

Levels of beneficial gut microbiota, specifically *Lactobacillus *and *Bifidobacterium*, were assessed in fecal samples from individuals with COVID-19, revealing a consistent reduction in these levels across all tested samples [14]. Lactobacilli are Gram-positive organisms that secrete lactic acid to inhibit toxic bacteria [22]. The number of gut commensals and *Bifidobacteria *was low throughout the intestinal tract and correlated with high concentrations of inflammatory cytokines. *Bifidobacterium *strains are considered probiotic microorganisms that can constitute up to 90% of the total bacterial population. Numerous studies have shown that the *Bifidobacterium *species can inhibit pathogens through the production of organic acids, antibacterial peptides, quorum-sensing inhibitors, or immune stimulation [18].

Changes in *Faecalibacterium prausnitzii *have been linked to dysbiosis in several human disorders [19]. It is the main butyrate producer found in the intestine, which has a crucial role in gut physiology. Butyrate reduces intestinal mucosa inflammation by inhibiting nuclear factor kappa B (NF-κB) transcription factor, upregulating peroxisome proliferator-activated receptor gamma (PPARγ), and inhibiting INF-γ [23]. Overall, inflammation and gut dysbiosis observed in COVID-19 have been associated with a decrease in the number of beneficial bacteria in the gut and an increase in bacteria linked to gut dysbiosis. It is speculated that the increased ratio of organ dysfunction might be associated with ICU-iatrogenically induced gut microbial diversity due to the increased use of antibiotics [24]. Additionally, patients taking antibiotics such as azithromycin, amoxicillin-clavulanate, cephalosporin, and tetracycline during COVID-19 have altered gut microbiota. This has been associated with the overgrowth of opportunistic pathogens such as *Bacteroides nordii*, *Actinomyces viscosus*, and *Clostridium hathewayi *[25].

**Figure 2:**
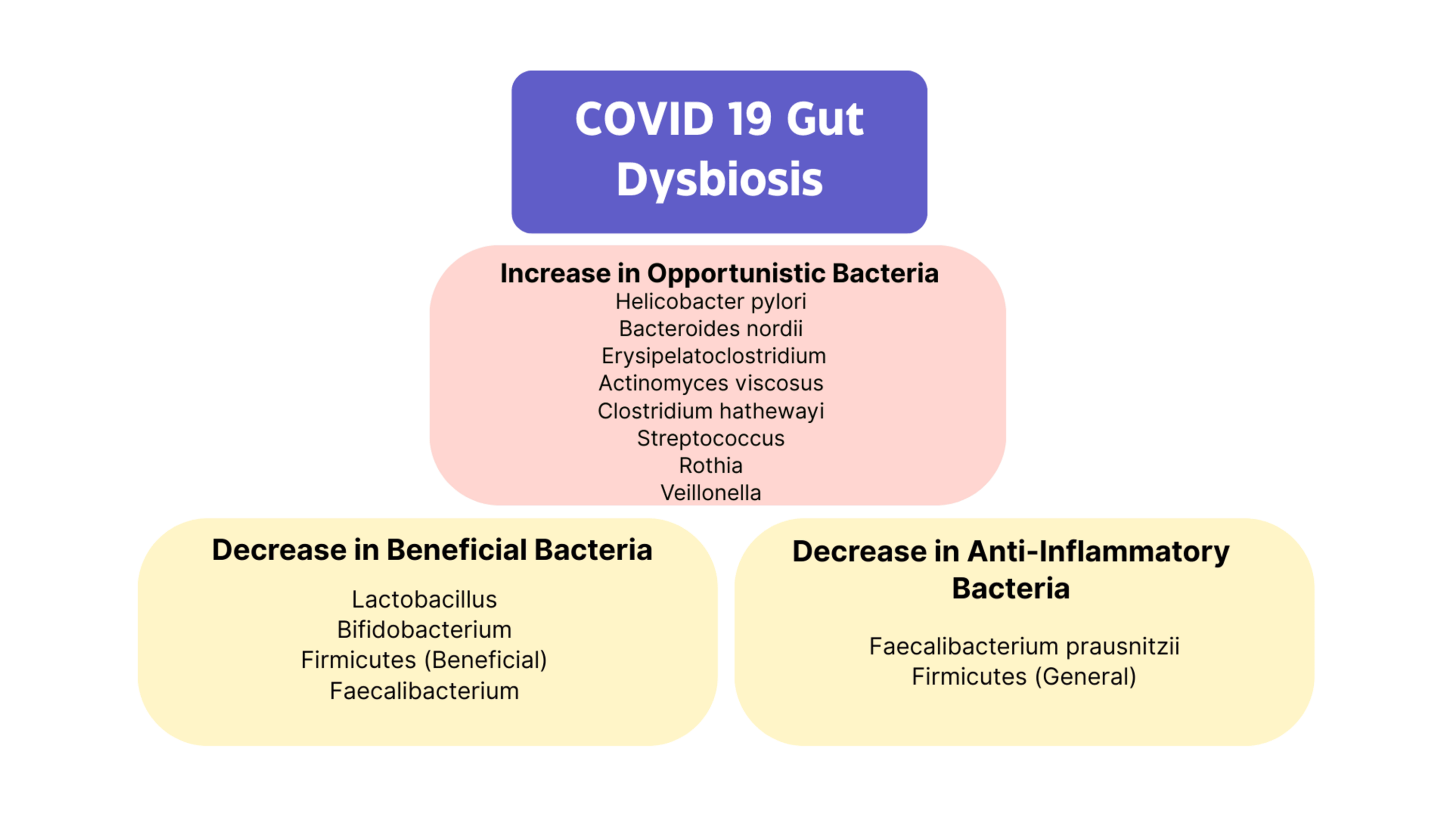
Various organisms composing the gut microbiota in COVID-19 patients. Image created with Mural

An increase in opportunistic pathogens was seen in COVID-19 patients compared to control group patients; these pathogens were *Streptococcus*, *Rothia*, *Veillonella*, *Erysipelatoclostridium*, and *Actinomyces*. These specific taxa were associated with a higher index of bacterial infection and elevated CRP. Figure 2 illustrates these microbial changes, categorizing the gut microbiota alteration in COVID-19 into increases in opportunistic and pro-inflammatory bacteria and decreases in beneficial and anti-inflammatory bacteria. This imbalance is thought to potentially compromise gut mucosa by promoting mucosal permeability, leading to increases in pathogenic bacteria and decreases in beneficial bacteria, creating inflammation, increased endotoxin, and cytokine release. This study suggested that the gut microbiome can have a potential value as a diagnostic biomarker and therapeutic target for COVID-19. It identified the following species as biomarkers for COVID-19: *Fusicatenibacter*, *Romboutsia*, *Intestinibacter*, *Actinomyces*, and *Erysipelatoclostridium*. These final biomarkers were chosen based on the most populated microbes in the gut of patients who had COVID-19 [16]. They may serve to diagnose and therapeutically target COVID-19 infection, since recognizing specific species may lead to more precise treatments of GI symptoms, such as targeted probiotic and prebiotic use.

Emerging Therapies

**Figure 3:**
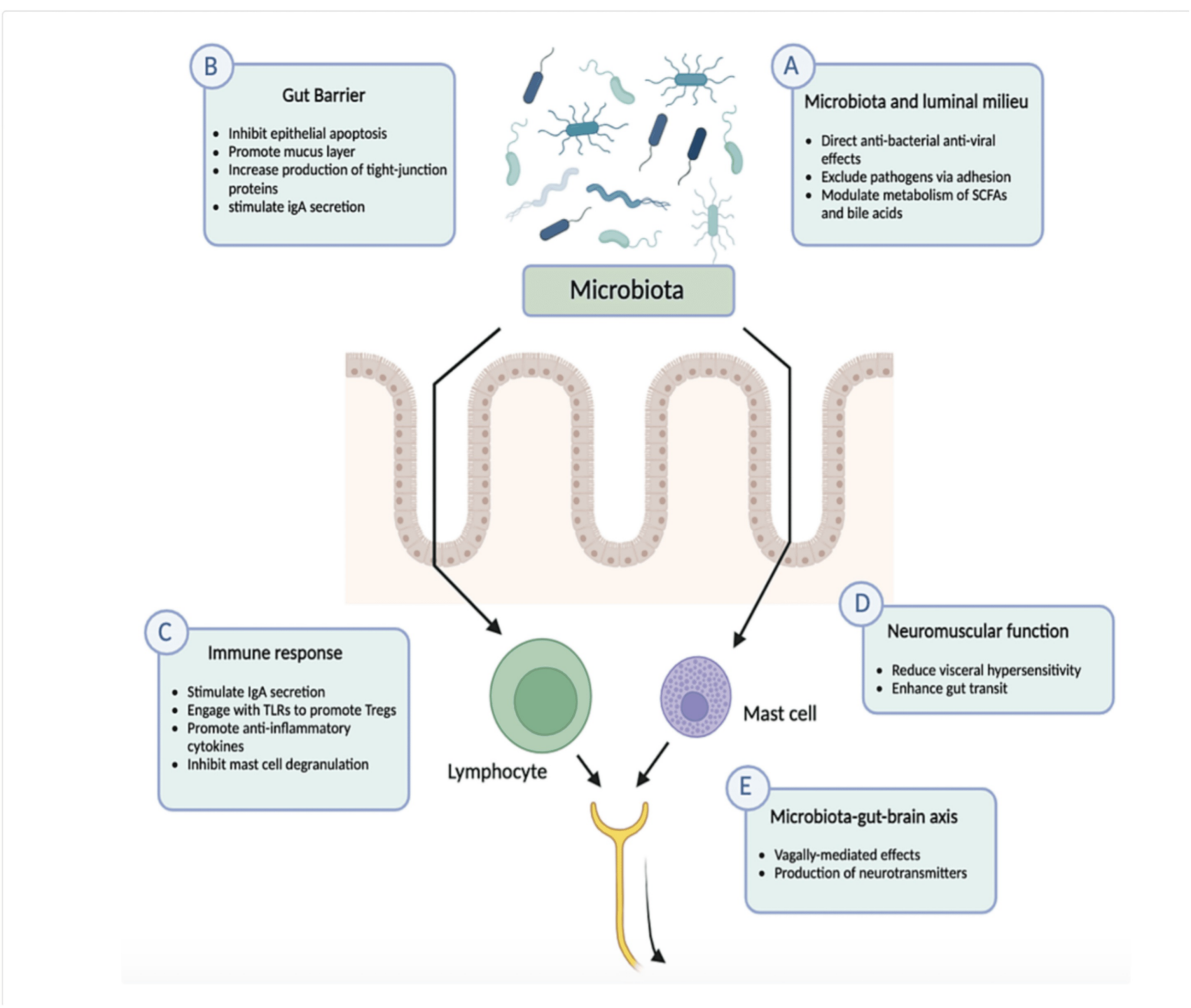
Possible mechanisms of action of probiotics. The figure depicts the possible mechanisms of action of probiotics, which include the microbiota and luminal milieu, gut barrier, immune response, neuromuscular function, and the microbiota–gut–brain axis. (A) Effects on the microbiota and luminal milieu. (B) Effects on the gut barrier. (C) Immune effects. (D) Effects on neuromuscular function. (E) Impact on the microbiota-gut-brain axis. IgA: immunoglobulin A; SCFA: short-chain fatty acid; TLR: Toll-like receptor; Treg: regulatory T lymphocyte. Image created with Biorender (www.biorender.com; BioRender, Toronto, Canada).

Probiotics are microorganisms that have beneficial properties for the host, while prebiotics are dietary components that promote the growth and metabolic activity of beneficial bacteria [26]. Many probiotics are derived from commensal bacteria present in a healthy human gut; they are composed mostly of live Gram-positive organisms [15]. The gut commensals predominantly aid in nutrient metabolism, drug metabolism, the prevention of colonization of pathogenic microorganisms, and in intestinal barrier function [17]. This allows probiotics to mimic these bacteria and establish homeostasis in the gut, as well as promoting intestinal barrier integrity and reducing inflammatory responses (Figure 3) [15,26]. Prebiotics are food ingredients that contain non-digestible oligosaccharides such as galactooligosaccharides and inulin. When they are combined with a probiotic, the resulting combination is termed a synbiotic [17].

Overall, these represent a combination of strategies that have been associated with improvements in gut microbiota composition in some patient populations. Specifically, they play a major role as treatments for patients with severe gut diseases and long-term use of antibiotics, antidepressants, statins, laxatives, and proton pump inhibitors [22].

In 19 clinical trials, the specific strains of probiotics used most commonly were in the *Lactobacilli *and *Bifidobacteria *genus due to the significant decrease during a COVID-19 infection as mentioned above [22]. There are over 250 species of *Lactobacilli, *which are beneficial in chronic inflammatory diseases such as obesity, diabetes, and hypertension. *Lactobacilli *and *Paenibacillus *may play a role in COVID-19 prevention and treatment by potentially binding to ACE2 and inhibiting SARS-CoV-2 attachment to host cells, as suggested in preclinical studies. *Lactobacillus fermentum *was proven in obese mouse models to decrease inflammatory markers, improve gut epithelial cell function, and increase the gut barrier [22]. *Lacticaseibacillus rhamnosus *has been proven to prevent the incidence of ventilator-associated pneumonia [27].

*Bifidobacteria *have been shown to eliminate viruses by increasing phagocytic activities in monocytes and killer cells. *Bifidobacterium bifidum *is a specific strain that significantly decreased IL-6, a proinflammatory cytokine seen in chronic diseases [22]. Sivomixx® (Ormendes SA, Jouxtens-Mézery, Switzerland) is a probiotic mixture of *Lactobacillus*, *Bifidobacteria*, and *Streptococcus *that has been used in multiple clinical trials with COVID-19 patients. In an observational cohort study involving 200 patients with severe COVID-19 pneumonia, treatment with Sivomixx® was associated with a reduced risk of death. In another study, almost all patients with COVID-19 given Sivomixx® reduced their gastrointestinal symptoms within 72 hours of treatment, in contrast to less than half in the control group [27].

Prebiotics such as fibers, inulin, fructan, glucans, and resistant starches can promote the growth of commensal bacteria by increasing the production of short-chain fatty acids such as butyrate and propionate [28]. These short-chain fatty acids increase the secretion of mucins and anti-microbial peptide defensins, promoting anti-inflammation [22]. Prebiotics increase microbial metabolites, which are essential for a healthy microbiome [26]. These commensal bacterial metabolites were found to be more effective at re-establishing a healthy gut microbiome in cases with severe intestinal COVID-19 infections and extensive antibiotic use, increasing commensal bacteria. Supplementation via these metabolites has been suggested to potentially reduce the severity of COVID-19 in acute infections and decrease gut dysbiosis in patients with previous infections [22].

Inulin has been proven to increase the amount of *Bifidobacterium *and *Lachnispiraceae*, which hold beneficial properties in the gut microbiome [22]. Fructo-Oligosaccharides (FOS) have also been proven to increase beneficial probiotic bacteria in the gut, such as *Bifidobacterium *and *Lactobacillus*, as well as butyrate-producing bacteria. There are five types of resistant starches that can only be digested by microorganisms in the human gut. Each of these microorganism strains produces specific beneficial properties, such as increasing *Bifidobacterium*, short-chain fatty acids, and butyrate [22]. The use of inulin, FOS, and resistant starches has been associated with improvements in gut microbiome balance in patients with inflammatory diseases, including those affected by COVID-19. 

Synbiotics provide both the gut microbe as well as the foundation for fermentation - the combination of a probiotic and prebiotic is more effective in modulating the gut microbiome. For example, *Lactobacillus *and tagatose were proven to work better together in inhibiting inflammatory effects rather than each individually. The synbiotic *Bacillus coagulans *and sugarcane fiber increased short-chain fatty acids and inhibited inflammatory effects in the gut [22]. A clinical trial using a synbiotic called OMNi-BiOTic® 10 AAD (OMNi-BiOTic, Port Chester, USA) was used for COVID treatment [29]. This synbiotic was composed of *Bifidobacteria *strains, one *Enterococcus *strain, and seven *Lactobacillus *strains combined with maize starch, inulin, and FOS. The study found that the synbiotic was linked to reduced inflammation, increased gut microbiome diversity, and improved the gut barrier [29].

Fecal microbiota transplantation (FMT) has been proven to help restore the gut microbiota for individuals infected with COVID-19 [30]. In a study, 11 positive COVID-19 patients who were discharged from the hospital within a 3-month period were chosen to undergo a trial of FMT. Each participant received a 4-day trial of 10 capsules each day. The oral capsules were prepared from a single donor. Abstinence from antibiotics for two weeks before the trial began was ensured. Fecal samples were obtained one week before and after the FMT treatment. Gut microbiome alterations and GI symptoms improved following the treatment, suggesting a potential therapeutic role for FMT. The amount of *Actinobacteria *and *Proteobacteria *was restored to the average level of the general population after FMT.

Both *Bifidobacteria *and *Faecalibacterium* are very dominant and are closely related to gut health. Both genera increased after FMT. In addition, before and after FMT treatment, 69 lymphocyte subsets were analyzed, revealing a marked effect on B lymphocytes. There was a decrease in naive B cells and an increase in memory B cells, as well as non-switched B cells, all evidence of favorable effects on the immune system [30].

Probiotics, prebiotics, synbiotics, and FMT are all crucial to the role of maintaining gut health and addressing gut dysbiosis, particularly for patients with inflammatory diseases such as COVID-19. Clinical trials have proven their benefits in terms of improving GI symptoms, gut microbiome, and inflammation. Collectively, these interventions highlight the therapeutic potential of gut microbiome modulation in scenarios of inflammatory diseases.

## Conclusions

Gastrointestinal distress associated with COVID-19 infection is a clinically significant syndrome due to the potential severity of symptoms, cellular injury, and long-term dysbiosis. ACE2 receptors located in the gastrointestinal and respiratory systems are used by COVID-19 to enter cells via the gut-lung axis. Upon entry, inflammation is induced, leading to cellular damage, cytokines, and direct damage to tight junctions, which may contribute to GI distress. GI alterations associated with COVID-19 infection have a strong correlation to microbiome composition and severity of symptoms. The gut microbiota plays an important role in modulating metabolic activities and controlling functions such as nutrient, drug, and xenobiotic metabolism, making up 50% of the cells found within the human body.

COVID-19 has been associated with a decrease in beneficial microbes such as Firmicutes and Bacteroides, and an increase in the opportunistic *H. pylori*, which has been linked to a pro-inflammatory state in the gut and contributes to more significant systemic alterations, including an increase in ACE2R expression, allowing COVID-19 to enter cells. Decreases in species such as *Lactobacillus *and *Bifidobacterium *have been associated with impaired immune responses. Probiotics may help buffer the dysbiosis that has been observed in association with COVID-19 infections. Preclinical studies suggest that they may bind to ACE2R, potentially limiting SARS-CoV-2 entry into cells, and may also be associated with decreased inflammatory markers, promote phagocytic activity by the immune system, and boost defenses against COVID-19.

Prebiotics can also be used to reestablish the balance between opportunistic and beneficial bacteria in the gut microbiome. Research shows that when combined as a synbiotic, pre- and probiotics can be even more effective. Additionally, FMT has been associated with improvements in immune markers and GI symptoms in patients recovering from COVID-19. Overall, these findings demonstrate the importance of prioritizing the microbiome when assessing patients with viral or inflammatory conditions. By incorporating the microbiome-focused strategies into treatment regimens, clinicians may support recovery, modulate inflammation, and improve outcomes in patients with viral or inflammatory conditions.

## References

[1] Cui J, Li F, Shi ZL (2019). Origin and evolution of pathogenic coronaviruses. Nat Rev Microbiol.

[2] Hamming I, Timens W, Bulthuis ML, Lely AT, Navis G, van Goor H (2004). Tissue distribution of ACE2 protein, the functional receptor for SARS coronavirus. A first step in understanding SARS pathogenesis. J Pathol.

[3] Jin X, Lian JS, Hu JH (2020). Epidemiological, clinical and virological characteristics of 74 cases of coronavirus-infected disease 2019 (COVID-19) with gastrointestinal symptoms. Gut.

[4] Alberca GG, Alberca RW (2021). Nutrition and the microbiota post-COVID-19. Saudi J Gastroenterol.

[5] Gutiérrez-Castrellón P, Gandara-Martí T, Abreu Y Abreu AT (2022). Probiotic improves symptomatic and viral clearance in Covid19 outpatients: a randomized, quadruple-blinded, placebo-controlled trial. Gut Microbes.

[6] Guo M, Tao W, Flavell RA, Zhu S (2021). Potential intestinal infection and faecal-oral transmission of SARS-CoV-2. Nat Rev Gastroenterol Hepatol.

[7] Luo X, Zhou GZ, Zhang Y, Peng LH, Zou LP, Yang YS (2020). Coronaviruses and gastrointestinal diseases. Mil Med Res.

[8] Aroniadis OC, DiMaio CJ, Dixon RE (2020). Current knowledge and research priorities in the digestive manifestations of COVID-19. Clin Gastroenterol Hepatol.

[9] Perisetti A, Goyal H, Gajendran M, Boregowda U, Mann R, Sharma N (2020). Prevalence, mechanisms, and implications of gastrointestinal symptoms in COVID-19. Front Med (Lausanne).

[10] Luo S, Zhang X, Xu H (2020). Don't overlook digestive symptoms in patients with 2019 novel coronavirus disease (COVID-19). Clin Gastroenterol Hepatol.

[11] Norouzi Masir M, Shirvaliloo M (2022). Symptomatology and microbiology of the gastrointestinal tract in post-COVID conditions. JGH Open.

[12] Lamers MM, Beumer J, van der Vaart J (2020). SARS-CoV-2 productively infects human gut enterocytes. Science.

[13] Zhang W, Du RH, Li B (2020). Molecular and serological investigation of 2019-nCoV infected patients: implication of multiple shedding routes. Emerg Microbes Infect.

[14] Xu H, Akinyemi IA, Chitre SA, Loeb JC, Lednicky JA, McIntosh MT, Bhaduri-McIntosh S (2022). SARS-CoV-2 viroporin encoded by ORF3a triggers the NLRP3 inflammatory pathway. Virology.

[15] DeGruttola AK, Low D, Mizoguchi A, Mizoguchi E (2016). Current understanding of dysbiosis in disease in human and animal models. Inflamm Bowel Dis.

[16] Gu S, Chen Y, Wu Z (2020). Alterations of the gut microbiota in patients with coronavirus disease 2019 or h1 n1 influenza. Clin Infect Dis.

[17] Jandhyala SM, Talukdar R, Subramanyam C, Vuyyuru H, Sasikala M, Nageshwar Reddy D (2015). Role of the normal gut microbiota. World J Gastroenterol.

[18] Hidalgo-Cantabrana C, Delgado S, Ruiz L, Ruas-Madiedo P, Sánchez B, Margolles A (2017). Bifidobacteria and their health-promoting effects. Microbiol Spectr.

[19] Miquel S, Martín R, Rossi O (2013). Faecalibacterium prausnitzii and human intestinal health. Curr Opin Microbiol.

[20] Khan M, Mathew BJ, Gupta P, Garg G, Khadanga S, Vyas AK, Singh AK (2021). Gut dysbiosis and IL-21 response in patients with severe COVID-19. Microorganisms.

[21] Balamtekin N, Artuk C, Arslan M, Gülşen M (2021). The effect of Helicobacter pylori on the presentation and clinical course of coronavirus disease 2019 infection. J Pediatr Gastroenterol Nutr.

[22] Chen J, Vitetta L (2021). Modulation of gut microbiota for the prevention and treatment of COVID-19. J Clin Med.

[23] Lopez-Siles M, Duncan SH, Garcia-Gil LJ, Martinez-Medina M (2017). Faecalibacterium prausnitzii: from microbiology to diagnostics and prognostics. ISME J.

[24] Din AU, Mazhar M, Waseem M (2021). SARS-CoV-2 microbiome dysbiosis linked disorders and possible probiotics role. Biomed Pharmacother.

[25] Kaźmierczak-Siedlecka K, Vitale E, Makarewicz W (2020). COVID-19 - gastrointestinal and gut microbiota-related aspects. Eur Rev Med Pharmacol Sci.

[26] Quigley EM (2019). Prebiotics and probiotics in digestive health. Clin Gastroenterol Hepatol.

[27] Hung YP, Lee CC, Lee JC, Tsai PJ, Ko WC (2021). Gut dysbiosis during COVID-19 and potential effect of probiotics. Microorganisms.

[28] Holscher HD (2017). Dietary fiber and prebiotics and the gastrointestinal microbiota. Gut Microbes.

[29] (2022). Synbiotic therapy of gastrointestinal symptoms during COVID-19 infection | Medical University of Graz. https://ctv.veeva.com/study/synbiotic-therapy-of-gastrointestinal-symptoms-during-covid-19-infection.

[30] Liu F, Ye S, Zhu X (2021). Gastrointestinal disturbance and effect of fecal microbiota transplantation in discharged COVID-19 patients. J Med Case Rep.

